# Current Diagnostic Practices for Sacroiliac Joint Pain Among Interventional Pain Physicians: Results of an Exploratory Practice Survey

**DOI:** 10.3390/diagnostics16213468

**Published:** 2026-10-23

**Authors:** Jamal Hasoon, Jenna Chuan, Grant H. Chen, Christopher L. Robinson, Omar Viswanath, Alan D. Kaye, Alaa Abd-Elsayed, Thomas T. Simopoulos, Jatinder Gill

**Affiliations:** 1 Department of Anesthesiology, Critical Care and Pain Medicine, The University of Texas Health Science Center at Houston, Houston, TX 77030, USA; 2 The University of Texas at Austin, Austin, TX 78712, USA; 3 Division of Pain Medicine, Department of Anesthesiology and Critical Care Medicine, Johns Hopkins University School of Medicine, Baltimore, MD 21205, USA; 4 Mountain View Headache and Spine Institute, Phoenix, AZ 85050, USA; 5 Department of Anesthesiology, Louisiana State University Health Shreveport, Shreveport, LA 71103, USA; 6 Department of Anesthesiology, University of Wisconsin School of Medicine and Public Health, Madison, WI 53792, USA; 7 Department of Anesthesiology, Critical Care, and Pain Medicine, Beth Israel Deaconess Medical Center, Harvard Medical School, Boston, MA 02215, USA

**Keywords:** sacroiliac joint, sacroiliac joint pain, diagnosis, diagnostic injection, low back pain, interventional pain medicine, chronic pain

## Abstract

**Background:** Sacroiliac (SI) joint pain contributes to chronic axial low back pain, but diagnosis remains challenging because no single historical feature, physical examination maneuver, imaging finding, or injection response is universally accepted as definitive. Although guidelines recommend multimodal assessment, less is known about how interventional pain physicians diagnose SI joint pain in real-world practice. **Objective:** We aim to describe self-reported diagnostic practices for suspected SI joint-mediated pain among interventional pain physicians. **Study Design:** Exploratory cross-sectional survey. **Methods:** An anonymous, voluntary SurveyMonkey questionnaire was distributed between 1 January 2026, and 20 June 2026, to physicians practicing interventional pain medicine through academic institutional email distribution, professional contacts, and social media platforms. The present analysis focused on survey items related to physician specialty, practice setting, diagnostic approach, discordance between history and physical examination, use of diagnostic injections, estimated prevalence of SI joint pain, and management when SI joint and lumbar facet pain coexist. **Results:** Forty-eight physicians initiated the survey, although some respondents skipped individual items; therefore, item-level response totals ranged from 47 to 48. Respondents were predominantly anesthesiologists (72.3%) and most commonly practiced in academic medical centers (58.3%). All incorporated history and physical examination into their primary diagnostic approach, and 62.5% also included diagnostic injection. A single diagnostic injection was reported by 59.6%, whereas 27.7% routinely used two or more. **Conclusion:** Respondents reported multimodal clinical assessment with variation in diagnostic injection strategies. This small, self-selected sample describes practice patterns and does not establish national consensus, diagnostic standards, or population prevalence.

## 1. Introduction

Sacroiliac (SI) joint pain is recognized as a clinically important contributor to chronic axial low back pain. The SI joint may be implicated in a substantial proportion of patients presenting with pain localized at or below the belt line [1,2,3]. The SI joint is a large diarthrodial articulation between the sacrum and ilium that transfers load between the spine and lower extremities. It contains opposing articular surfaces, a synovial space, and a fibrous capsule, but permits relatively little motion because of its complementary, multifaceted joint architecture. Innervation is complex and variable, which may contribute to heterogeneous and referred pain patterns. Posterior innervation is primarily derived from the L5 dorsal ramus and lateral branches of the S1-S3 dorsal rami, with possible contributions from the L4 medial branch and S4 lateral branch, while anterior innervation may arise from the L5-S2 ventral rami with variable L4 contribution [2,3,4]. Estimates suggest that the SI joint accounts for approximately 15–30% of chronic, non-radicular low back pain predominantly below L5 [1,2,3]. Certain populations carry heightened risk, including patients with prior lumbar or lumbosacral fusion, in whom prevalence estimates increase substantially, as well as those with altered pelvic biomechanics, trauma, pregnancy-related ligamentous laxity, leg-length discrepancy, or coexisting degenerative spinal pathology [1,2,3,4]. Despite growing clinical awareness, establishing a definitive diagnosis of SI joint-mediated pain remains challenging.

The diagnostic difficulty arises because no single clinical feature or examination maneuver can reliably confirm the SI joint as the pain generator. Historical clues such as pain below the posterior superior iliac spine, buttock-predominant symptoms, and pain provoked by transitional movements or load transfer may suggest SI joint involvement but overlap substantially with lumbar facetogenic pain, discogenic pain, hip pathology, myofascial syndromes, and radiculopathies [1,5]. Provocative physical examination maneuvers, including the Patrick/FABER, Gaenslen, thigh thrust, sacral thrust, distraction, and compression tests, are widely employed; however, individual tests demonstrate variable sensitivity and specificity [6,7]. Imaging modalities such as radiography, CT, and MRI may reveal degenerative or inflammatory changes but have low sensitivity for mechanical SI joint pain and correlate poorly with symptoms [1,8,9,10]. Consequently, image-guided diagnostic SI joint injections with local anesthetic serve as the current reference standard for confirming SI joint-mediated pain, despite inherent limitations, including potential false positives with single uncontrolled injections [2]. The optimal number of diagnostic injections and the threshold for a positive response remain debated; dual controlled blocks with higher pain-relief thresholds (e.g., 70–75%) may improve specificity relative to more permissive single-block paradigms, although the appropriate threshold depends on the intended diagnostic or treatment-selection purpose [1,2,11,12].

Contemporary clinical practice, therefore, relies on a multimodal diagnostic framework that integrates clinical history, physical examination findings, imaging when indicated, and response to diagnostic injections. However, guideline recommendations and real-world clinical practice may not always align. Understanding how interventional pain physicians actually approach the diagnosis of SI joint pain is clinically important, as variability in diagnostic methods may influence patient selection for downstream treatments, including therapeutic SI joint injections, sacral lateral branch radiofrequency ablation, and minimally invasive SI joint fusion. Published diagnostic studies and guidelines primarily address test performance and recommended pathways; less is known about physicians’ self-reported use of these approaches and the diagnostic uncertainty they encounter in routine practice.

The objective of this study was to describe self-reported diagnostic practices in an exploratory sample among interventional pain physicians for suspected SI joint-mediated pain. Specifically, this survey examined primary diagnostic strategies employed, perceived discordance between clinical history and physical examination findings, use of single versus serial diagnostic injections, estimated prevalence of SI joint pain among patients with chronic axial pain, and treatment prioritization when SI joint and lumbar facet pain coexist. The principal question was how respondents combine clinical assessment with diagnostic injections, including single versus serial injection strategies; the remaining items provide context for these decisions.

## 2. Methods

### 2.1. Study Design and Survey Distribution

This was an anonymous, exploratory cross-sectional survey administered using SurveyMonkey between 1 January 2026, and 20 June 2026. Distribution occurred through email to academic institutions, professional contacts, and social media outlets relevant to interventional pain medicine, including open electronic links. The exact number of physicians who received or viewed the questionnaire could not be determined; therefore, an exact response rate could not be calculated. Respondents’ geographic distribution could not be established from the anonymous responses, and national representativeness was not demonstrated. Institutional Review Board approval was obtained from The University of Texas Health Science Center at Houston (HSC-MS-25-1110).

Eligible participants were practicing interventional pain physicians who perform SI joint interventions. Eligibility was not restricted to a particular medical specialty. Residents and fellows were excluded, and physicians who did not perform interventional procedures were requested not to participate. Participation was voluntary, no incentives were provided, and participants could exit at any time. All survey items were optional.

No target sample size was prespecified. The survey was exploratory and descriptive, and no formal power calculation was performed. Survey closure was guided by a decline in incoming responses. All eligible responses collected during the study period were included, including available answers from incomplete questionnaires.

SurveyMonkey supports restrictions on multiple submissions by browser or email invitation, depending on the collector type and configuration. However, since the survey was also distributed through open electronic links, repeat participation through different browsers or devices cannot be excluded. Potential duplicate submissions therefore remain a limitation and may have affected the reported response frequencies and percentages.

### 2.2. Survey Items Included in the Analysis

The present manuscript focused specifically on survey items related to SI joint diagnosis and diagnostic decision-making. Questions related to specific treatment modalities, including injection technique, radiofrequency ablation, and SI joint fusion, were collected as part of the broader survey but were not included in this analysis and will be reported separately. Demographic data, including physicians’ primary specialties and practice settings, were also collected and are presented to contextualize the diagnostic findings. The questionnaire was newly developed by three interventional pain physicians rather than adapted from a validated instrument. Five pain physicians from different institutions and a variety of practice settings pilot-tested the questionnaire for clarity before it was finalized. Frequency categories such as “frequently,” “occasionally,” and “rarely” were not quantitatively defined and reflect respondents’ subjective interpretations. The complete questionnaire, including all 36 questions and their response options, is provided in Supplementary Table S1.

Table 1 lists the survey questions and answer choices included in this diagnostic-focused analysis.

### 2.3. Statistical Analysis

Descriptive statistics were used to summarize survey responses. Percentages were calculated using the number of respondents who completed each item as the denominator. Skipped items were excluded only from the denominator of the relevant question; missing answers were not imputed. Thus, item-level denominators were 47 or 48, and skipped-response counts are identified in the tables and figure legends. No inferential comparisons were performed because the analysis was intended to describe an exploratory sample. Analyses were performed using built in SurveyMonkey tools and confirmed manually by the authors.

## 3. Results

### Respondent Characteristics

A total of 47–48 physicians completed the survey items included in this analysis. Among respondents who reported their specialty, most identified anesthesiology as their primary specialty (34/47, 72.3%), followed by physical medicine and rehabilitation (9/47, 19.2%) and neurology (4/47, 8.5%). No respondents identified radiology, emergency medicine, psychiatry, or another specialty.

Respondents most commonly practiced in academic medical centers (28/48, 58.3%). Other practice settings included hospital-based practice (9/48, 18.8%), private practice (8/48, 16.7%), multispecialty group practice (1/48, 2.1%), and other practice settings (2/48, 4.2%). Both physicians who reported practicing in an “other” setting indicated that they worked within a Veterans Affairs (VA) healthcare system.

Table 2 summarizes physicians’ specialties and primary practice settings. Percentages were calculated using completed responses for each item. Of 48 respondents, one skipped the specialty item and none skipped the practice-setting item.

**Figure 1:**
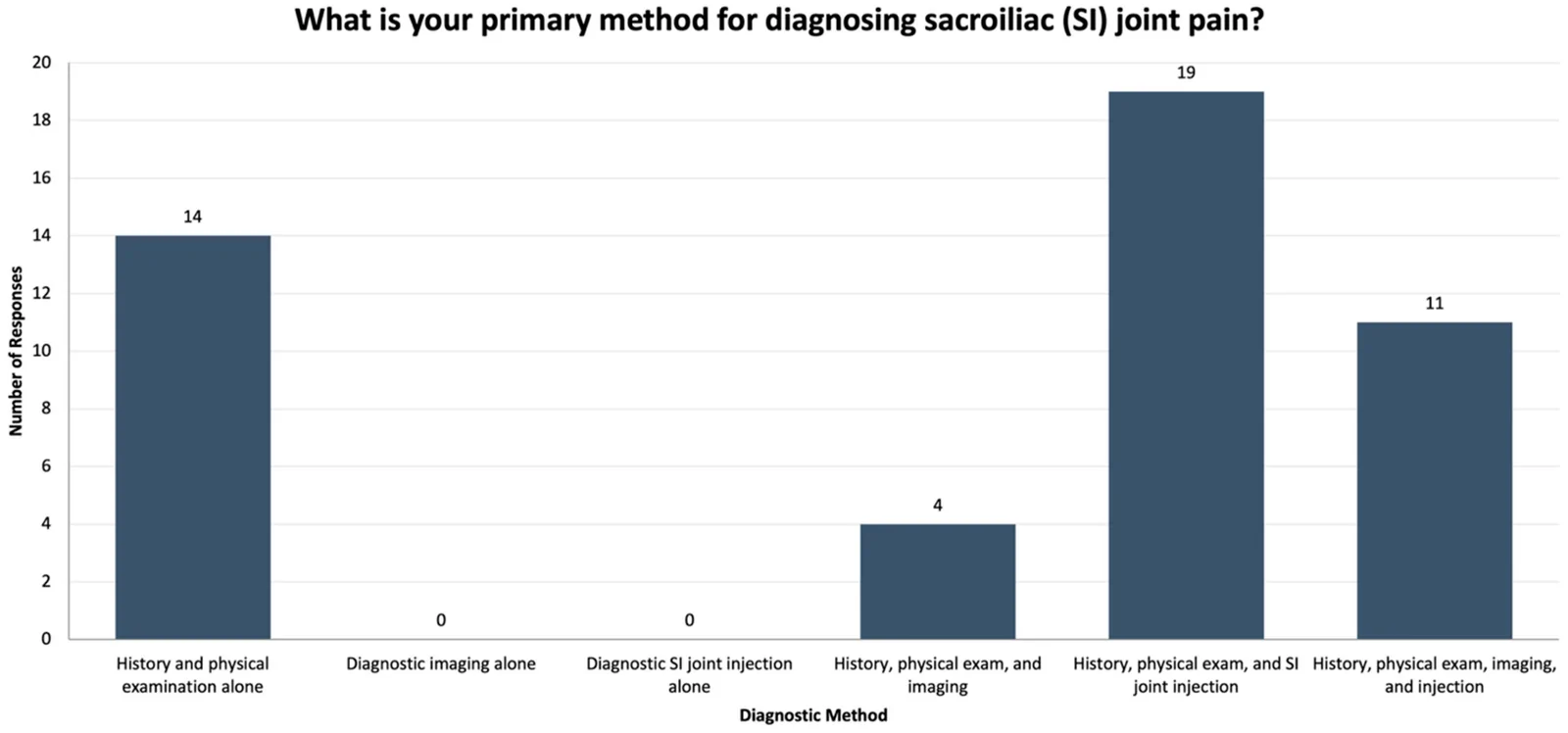
Primary method for diagnosing SI joint pain. Completed responses: *n* = 48; skipped responses: *n* = 0. Percentages use completed responses as the denominator.

When evaluating the primary method used to diagnose SI joint pain, 39.58% of physicians (19 out of 48) reported using a combination of history, physical examination, and diagnostic SI joint injection. In comparison, 29.17% (14 of 48) reported using only history and physical examination. Another 22.92% of respondents reported using history, physical examination, diagnostic imaging, and diagnostic SI joint injection (11 out of 48), and 8.33% reported using history, physical examination, and diagnostic imaging without an injection (four out of 48). No physicians reported diagnosing SI joint pain with imaging alone or with a diagnostic injection alone, and no physicians reported that they do not treat SI joint pain. These results demonstrate that all responding physicians use history and physical examination as part of their diagnostic approach, with a majority also using a diagnostic injection (62.5%, 30 out of 48) and a minority using diagnostic imaging (31.25%, 15 out of 48). The results are displayed in Figure 1.

**Figure 2:**
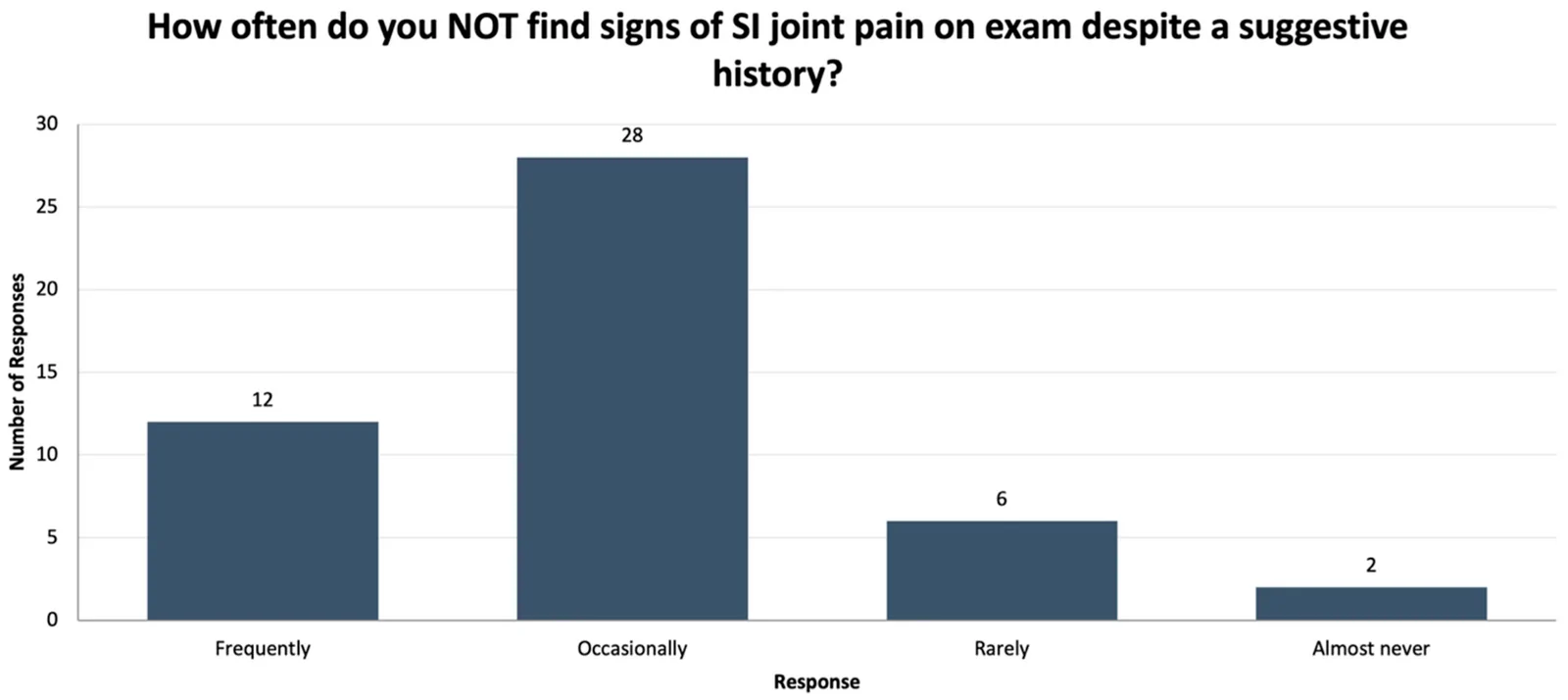
Frequency of absent examination findings despite a suggestive history. Completed responses: *n* = 48; skipped responses: *n* = 0. Percentages use completed responses as the denominator.

When assessing how often physicians do not find signs of SI joint pain on examination despite a suggestive history, 58.33% (28 out of 48) reported this occurs occasionally. In comparison, 25% (12 out of 48) reported this occurs frequently. Another 12.5% of respondents reported that this occurs rarely (six out of 48), and only 4.17% reported this almost never occurs (two out of 48). These results demonstrate that a majority of physicians encounter patients with a suggestive history but no signs of SI joint pain on examination, with 83.33% (40 out of 48) reporting this occurs at least occasionally. The results are displayed in Figure 2.

**Figure 3:**
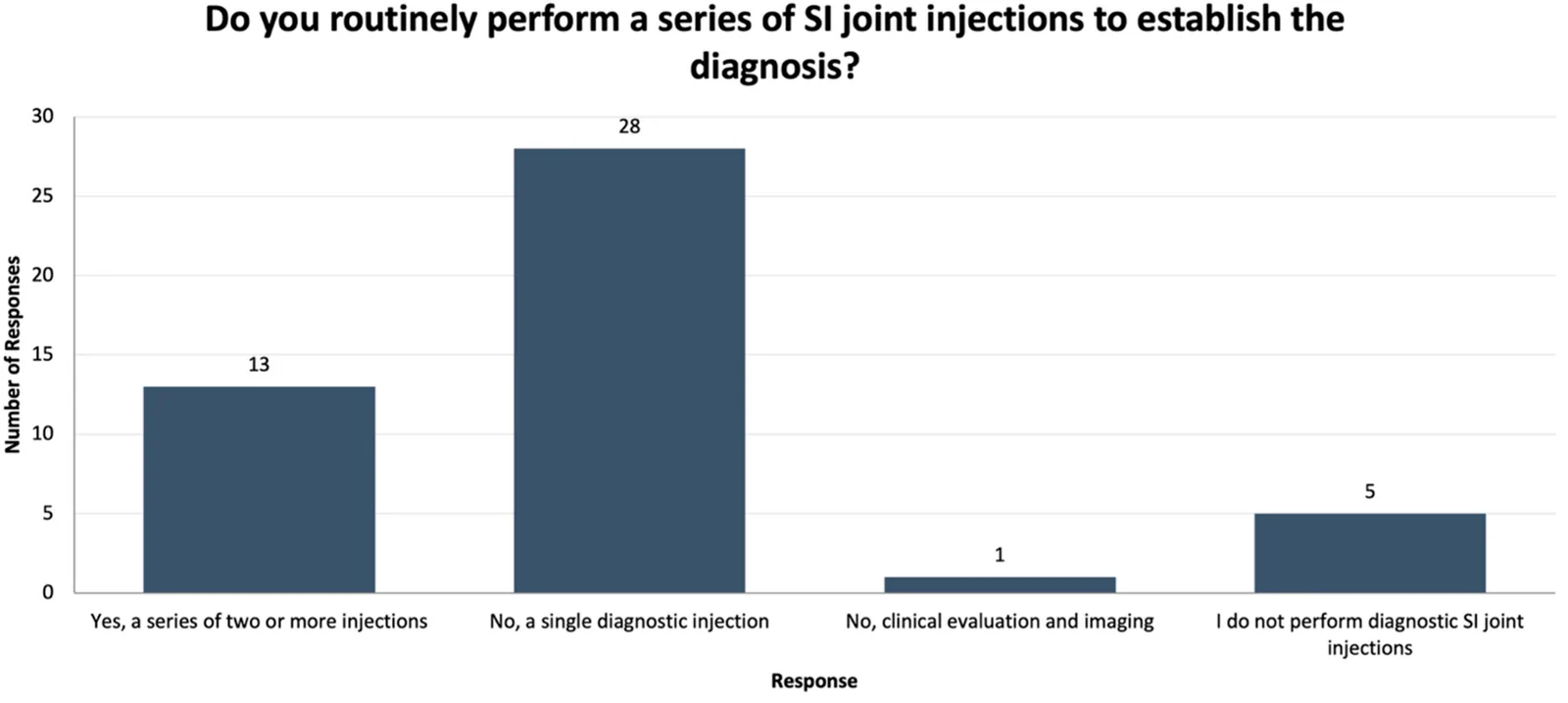
Use of single versus serial diagnostic SI joint injections. Completed responses: *n* = 47; skipped responses: *n* = 1. Percentages use completed responses as the denominator.

When evaluating the use of diagnostic injections to establish the diagnosis of SI joint-related pain, 59.57% of physicians (28 out of 47) reported using a single diagnostic injection, whereas 27.66% (13 out of 47) reported routinely performing a series of two or more injections. Another 10.64% of respondents reported that they do not perform diagnostic SI joint injections (five out of 47), and 2.13% reported relying on clinical evaluation and imaging instead (one out of 47). These results demonstrate that a majority of physicians perform diagnostic SI joint injections (87.23%, 41 out of 47), with most using a single injection rather than a series. Of note, only 47 physicians completed this question, with 1 physician skipping this question. The results are displayed in Figure 3.

**Figure 4:**
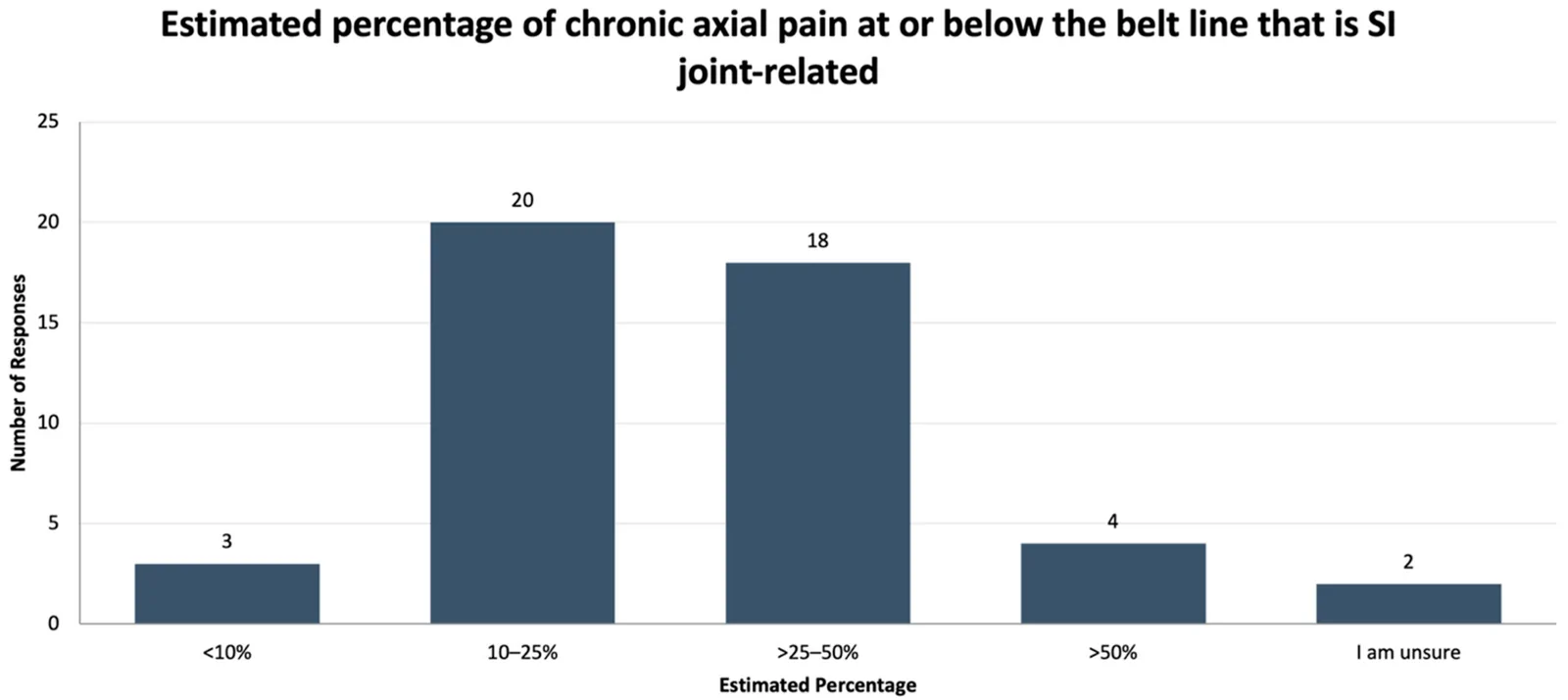
Physician-estimated prevalence of SI joint-related pain among patients with chronic axial pain at or below the belt line. Completed responses: *n* = 47; skipped responses: *n* = 1. Percentages use completed responses as the denominator.

When examining the estimated proportion of patients with chronic axial pain at or below the belt line whose pain is SI joint-related, 42.55% of physicians (20 out of 47) estimated 10% to 25%, while 38.3% (18 out of 47) estimated greater than 25% to 50%. Another 8.51% of respondents estimated greater than 50% (four out of 47), and 6.38% estimated less than 10% (three out of 47). Only 4.26% of respondents reported they were unsure (two out of 47). These responses describe physician-estimated prevalence within their own practices; a majority of respondents estimated that SI joint pathology accounts for 10% to 50% of patients with chronic axial pain at or below the belt line (80.85%, 38 out of 47). Of note, only 47 physicians completed this question, with 1 physician skipping it. The results are displayed in Figure 4.

**Figure 5:**
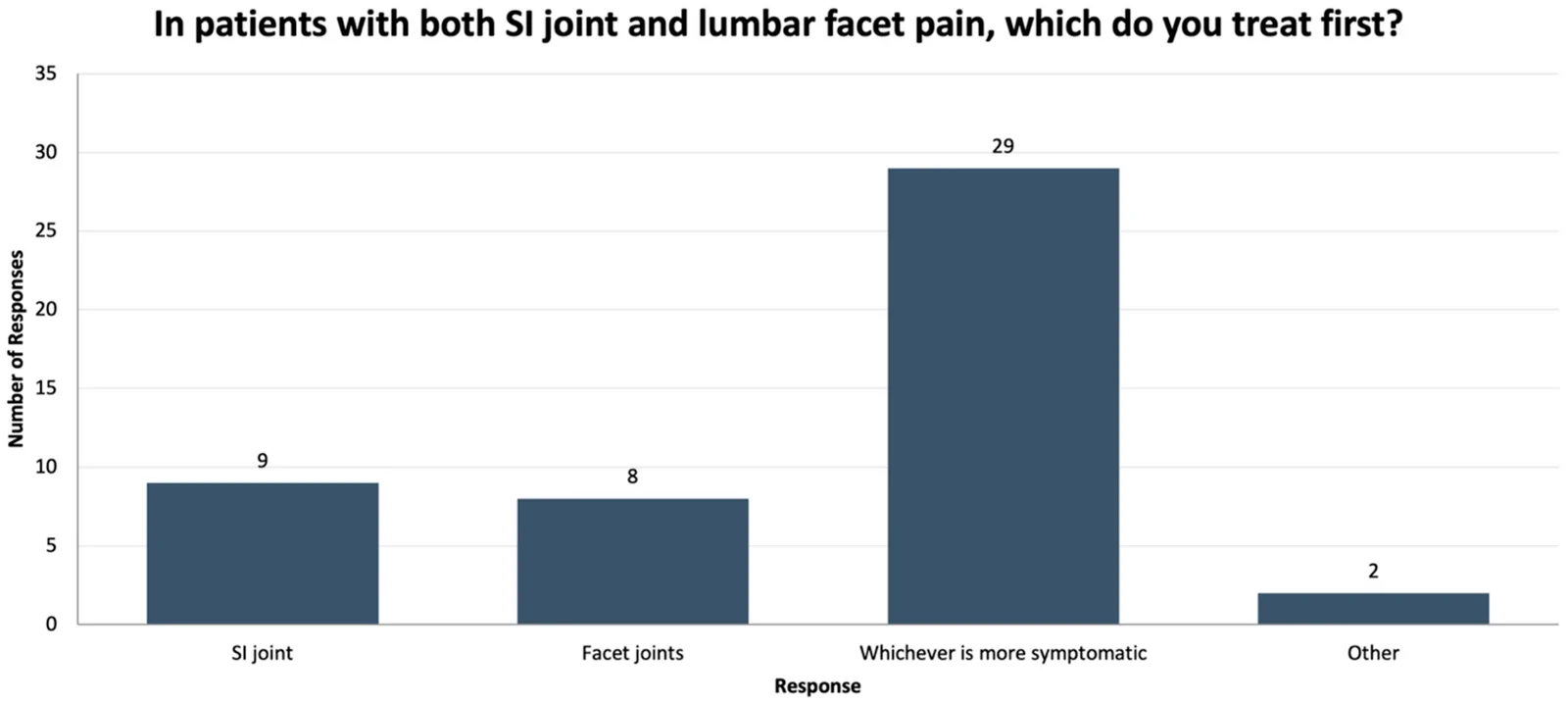
Treatment order when SI joint and lumbar facet pain coexist. Completed responses: *n* = 48; skipped responses: *n* = 0. Percentages use completed responses as the denominator.

In patients with both SI joint and lumbar facet pain, 60.42% of physicians (29 out of 48) reported treating whichever pain generator is more symptomatic. Another 18.75% of respondents reported treating the SI joint first (nine out of 48), while 16.67% reported treating the facet joints first (eight out of 48). Only 4.17% of respondents selected other (two out of 48). These results demonstrate that a majority of physicians base treatment sequencing on symptom severity rather than a fixed order, with the remaining respondents almost evenly divided between treating the SI joint and the facet joints first. The results are displayed in Figure 5.

## 4. Discussion

This exploratory practice survey describes how respondents approach the diagnosis of suspected SI joint-mediated pain. Several findings were notable. First, every respondent incorporated history and physical examination into the diagnostic process, and most physicians supplemented the clinical evaluation with a diagnostic SI joint injection. Second, discordance between a suggestive history and the physical examination was common, with over 80% of respondents reporting that this occurs at least occasionally. Third, although most physicians use diagnostic injections, practice varies on whether a single injection or a series of injections is used. Finally, most respondents estimated that SI joint pathology accounts for 10% to 50% of chronic axial pain at or below the belt line and generally prioritized treatment according to the pain generator that appeared most symptomatic. The principal finding is variation in diagnostic injection strategies within a shared reliance on clinical assessment in this sample. These self-reported patterns do not establish national consensus or validate a particular diagnostic pathway [1,2,11,12,13].

### 4.1. History and Physical Examination

The finding that all respondents relied on history and physical examination is consistent with the longstanding view that SI joint pain cannot be established by any single historical feature or examination maneuver [13,14,15]. Pain localization can be clinically useful; classic descriptions such as the Fortin finger test and SI joint pain referral maps demonstrated that characteristic buttock and posterior pelvic pain patterns may help identify patients in whom the SI joint should be considered [12,13,14,16]. However, SI joint pain may also refer to the lower lumbar region, groin, lower extremity, or even the foot, and this overlap can complicate differentiation from facetogenic, discogenic, hip, or radicular pain [15,17,18]. Physical examination has similar limitations. Individual maneuvers are imperfect, and false-positive findings can occur even in asymptomatic individuals [6,7,19]. Composite provocation testing improves diagnostic utility compared with reliance on a single maneuver. However, evidence suggests that negative test clusters may be more useful for ruling out SI joint pain than positive clusters are for definitively ruling it in [6,7,19]. The high frequency of history-examination discordance reported in the present survey may reflect diagnostic complexity, although the survey did not assess examination accuracy or the reasons for this discordance.

### 4.2. Imaging

Imaging also appears to function primarily as an adjunct rather than a stand-alone diagnostic test among respondents. Only 31.25% of respondents incorporated imaging into their primary diagnostic approach, and no physician reported using imaging alone. This is consistent with prior literature demonstrating that degenerative or inflammatory-appearing SI joint findings can be present in asymptomatic individuals and that imaging abnormalities do not necessarily identify the symptomatic pain generator [1,2,8,9,10,20]. SI joint dysfunction itself is typically associated with few abnormal imaging findings [1,2]. Imaging remains important when inflammatory sacroiliitis, fracture, infection, tumor, major structural abnormality, or another competing diagnosis is suspected, but mechanical SI joint-mediated pain generally requires correlation with the clinical presentation [1,2,8,9,10,20].

### 4.3. Diagnostic Injections

Diagnostic injections remain a central component of SI joint evaluation, as reflected by the 87.23% of respondents who reported performing diagnostic SI joint injections. Importantly, no respondent reported using an injection alone as the primary diagnostic method; rather, injections were generally integrated with the history and examination. This is consistent with the concept that image-guided local anesthetic blocks can provide confirmatory information but should be interpreted within the broader clinical context [1,2,3,12,13,14]. Diagnostic blocks themselves are not without limitations. Intra-articular injection primarily assesses intra-articular pain and may not fully capture extra-articular components of the SI joint complex (e.g., dorsal ligaments). At the same time, anesthetic spread and other technical factors may contribute to false-positive or false-negative responses [1,2]. These considerations may help explain why practice remains heterogeneous within this sample; the survey did not test diagnostic accuracy.

### 4.4. Single Versus Serial Blocks

The most apparent area of variation was the number of diagnostic injections used. Approximately 59.57% of respondents reported using a single diagnostic injection, whereas 27.66% routinely used a series of two or more injections. Earlier diagnostic literature emphasized controlled or comparative blocks to improve specificity, particularly because single uncontrolled injections may yield false-positive results [11]. At the same time, contemporary consensus recommendations acknowledge that the ideal block paradigm and response threshold remain debated and may differ according to whether the injection is used to identify intra-articular pain, select patients for radiofrequency treatment, or evaluate candidacy for fusion [1,2]. The present findings suggest that this debate may be reflected in respondents’ reported practice. The variability may represent differing interpretations of an evolving evidence base, local payer requirements, procedural goals, and physicians’ experience. These explanations were not directly assessed by the survey.

### 4.5. Estimated Prevalence

Physician-estimated prevalence reflects perceptions within respondents’ practices, not epidemiologically measured prevalence. More than 80% of respondents estimated that SI joint pathology accounts for between 10% and 50% of patients with chronic axial pain at or below the belt line. Reviews and controlled diagnostic studies have generally reported SI joint pain in the range of approximately 15% to 30% of patients with predominantly axial low back pain. However, prevalence varies substantially depending on the population studied and the diagnostic criteria used [1,2,3,11,12,13,14]. Higher rates may be encountered in selected postoperative populations, particularly after lumbar fusion, where altered biomechanics can increase stress across the SI joint [21,22]. The broad survey categories and absence of patient-level diagnostic verification preclude direct comparison with these published prevalence estimates.

### 4.6. Managing Coexisting Pain Generators

The management of coexisting pain generators further illustrates the individualized nature of real-world decision-making. When both SI joint and lumbar facet pain were present, 60.42% of physicians reported treating whichever pain generator appeared more symptomatic. In contrast, smaller and nearly equal proportions routinely treated the SI joint or facet joints first. This finding is clinically intuitive because multiple potential sources of axial pain frequently coexist, and the dominant pain generator may vary between patients and over time [1,2,23]. A symptom-driven approach may also allow clinicians to evaluate the response to one intervention before proceeding to treatment of an additional suspected source. Future studies could examine whether specific sequencing strategies improve outcomes when SI joint pain and lumbar facetogenic pain coexist.

Collectively, these responses describe reliance on history and physical examination with variation in the use of single versus serial diagnostic injections. They identify questions for larger, more representative surveys and prospective studies, but do not establish the effectiveness of a diagnostic strategy or the extent of guideline adherence. Interpretation is limited to this small, self-selected sample.

## 5. Limitations

This study has several limitations. The number of respondents was modest, with 47 to 48 physicians completing the diagnostic items, and the exact survey response rate is unknown because the survey was distributed through multiple academic, professional, and open electronic channels. As a result, the degree to which respondents represent the broader population of interventional pain physicians cannot be determined. Respondents were also predominantly anesthesiologists and physicians practicing in academic medical centers, which may influence the observed practice patterns. In addition, responses reflect self-reported practice rather than direct observation of clinical behavior, and one response was missing for each of selected items. Nonprobability sampling, self-selection, and an unverified geographic distribution limit generalizability. The questionnaire was pilot-tested for clarity, though undefined frequency categories may have been interpreted differently. Physician-estimated prevalence was not verified against patient-level data. Although SurveyMonkey supports restrictions on repeat submissions by browser or email invitation, duplicate submissions through open links accessed using different browsers or devices remain possible.

The findings should therefore be interpreted as an exploratory description of respondents’ practice patterns. The sample included several specialties and practice environments, but its size and composition preclude conclusions about national consensus or diagnostic standards.

## 6. Conclusions

In this exploratory survey, respondents incorporated history and physical examination into SI joint assessment, while diagnostic injection strategies varied, particularly the use of single versus serial injections. This is the principal clinical message of the analysis. The small, sample, predominance of anesthesiologists and academic physicians, and unverified geographic representation limit generalizability. These findings identify areas of practical consensus as well as opportunities for continued refinement of diagnostic pathways. This study is important because it highlights contemporary real-world data from practicing interventional pain physicians in an area where diagnostic evidence and recommendations continue to evolve, and it may help inform future efforts toward practical standardization.

## Figures and Tables

**Table 1:** Survey Questions and Response Options Included in the Present Analysis.

Survey Domain	Survey Question	Response Options
Physician specialty	What is your primary medical specialty?	Anesthesiology; Physical Medicine and Rehabilitation; Neurology; Radiology; Emergency Medicine; Psychiatry; Other
Practice setting	What is your primary practice setting?	Academic medical center; Private practice; Hospital-based practice; Multispecialty group practice; Other
Diagnostic approach	What is your primary method for diagnosing sacroiliac joint pain?	History and physical examination alone; Diagnostic imaging alone; Diagnostic SI joint injection with local anesthetic alone; History, physical examination, and diagnostic imaging; History, physical examination, and diagnostic SI joint injection; History, physical examination, diagnostic imaging, and diagnostic SI joint injection; Other; I do not treat SI joint pain
History and physical examination concordance	How often do you not find signs of SI joint pain on examination despite a suggestive history?	Frequently; Occasionally; Rarely; Almost never
Diagnostic injection strategy	Do you routinely perform a series of SI joint injections to establish the diagnosis of SI joint-related pain?	Yes, typically a series of two or more injections; No, I use a single diagnostic injection; No, I rely on clinical evaluation and imaging; I do not perform diagnostic SI joint injections
Estimated prevalence	In your patient population with chronic axial pain at or below the belt line, what percentage do you estimate is SI joint-related?	<10%; 10–25%; >25–50%; >50%; I am unsure
Coexisting pain generators	In patients with both SI joint and lumbar facet pain, which do you treat first?	SI joint; Facet joints; Whichever is more symptomatic; Other

**Table 2:** Respondent Characteristics.

Characteristic	Responses, *n* (%)
**Primary medical specialty (*n* = 47; skipped = 1)**	
Anesthesiology	34 (72.3%)
Physical Medicine and Rehabilitation	9 (19.2%)
Neurology	4 (8.5%)
Radiology	0 (0.0%)
Emergency Medicine	0 (0.0%)
Psychiatry	0 (0.0%)
Other	0 (0.0%)
**Primary practice setting (*n* = 48; skipped = 0)**	
Academic medical center	28 (58.3%)
Hospital-based practice	9 (18.8%)
Private practice	8 (16.7%)
Multispecialty group practice	1 (2.1%)
Other/VA	2 (4.2%)

## Data Availability

The original contributions presented in this study are included in the article. Further inquiries can be directed to the corresponding author. The complete questionnaire, including all 36 questions and their response options, is provided in Supplementary Table S1.

## Supplementary Materials

The following supporting information can be downloaded at https://www.mdpi.com/article/10.3390/diagnostics16213468/s1. Table S1: Complete Questionnaire. Survey on Practice Styles for Treating Sacroiliac (SI) Joint Pain in Interventional Pain Medicine.
