# Supplementary material for: Current Diagnostic Practices for Sacroiliac Joint Pain Among Interventional Pain Physicians: Results of an Exploratory Practice Survey
Source: Diagnostics (Basel). 2026 Oct 23;16(21):3468. doi: 10.3390/diagnostics16213468 (PMC13761591; doi:10.3390/diagnostics16213468)
Supplement: Supplementary file 1 [file diagnostics-16-03468-s001.zip › diagnostics-4545998-supplementary.pdf]

Supplementary Table S1

Complete Questionnaire

Survey on Practice Styles for Treating Sacroiliac (SI) Joint Pain in Interventional Pain Medicine

No.	Question	Answer choices
1	What is your primary medical specialty?	Anesthesiology<br>Physical medicine and rehabilitation<br>Neurology<br>Radiology<br>Emergency medicine<br>Psychiatry<br>Other (please specify)
2	What is your primary practice setting?	Academic medical center<br>Private practice<br>Hospital-based practice<br>Multispecialty group practice<br>Other (please specify)
3	What is your primary method for diagnosing sacroiliac (SI) joint pain? (Select one)	I do not treat SI joint pain<br>History and physical exam alone<br>Diagnostic imaging (X-ray, MRI, CT) alone<br>Diagnostic SI joint injection with local anesthetic alone<br>History, physical exam, and diagnostic imaging<br>History, physical exam, and diagnostic SI joint injection<br>History, physical exam, diagnostic imaging, and diagnostic SI joint injection<br>Other (please specify)
4	How often do you NOT find signs of SI joint pain on exam despite a suggestive history? (Select one)	Frequently<br>Occasionally<br>Rarely<br>Almost never
5	What percentage of pain relief do you consider clinically meaningful in diagnosing SI joint pain following diagnostic injection? (Select one)	I do not perform diagnostic SI joint injections<br>≥75% pain relief<br>≥50% pain relief<br>I do not use a percentage-based cutoff<br>Other (please specify)
6	Do you perform provocative maneuvers or repeat physical exam testing after a diagnostic SI joint block to assess relief? (Select one)	Yes, always<br>Yes, in most cases<br>Only in select patients<br>No, I rely solely on patient-reported relief<br>I do not perform diagnostic SI joint blocks
7	Do you routinely perform a series of SI joint injections to establish the diagnosis of SI joint-related pain? (Select one)	Yes, typically a series of 2 or more injections<br>No, I use a single diagnostic injection<br>No, I rely on clinical evaluation and imaging<br>I do not perform diagnostic SI joint injections
8	In your patient population with chronic axial pain at or below the belt line, what percentage do you estimate is SI joint-	<10%<br>10–25%<br>>25–50%

	related? (Select one)	>50%<br>I am unsure
9	How often do you perform fluoroscopically guided SI joint steroid injections? (Select one)	Frequently (multiple times per week)<br>Occasionally (a few times per month)<br>Rarely (a few times per year)<br>I do not perform SI joint injections
10	Do you routinely use contrast to confirm intra-articular placement? (Select one)	Yes, always<br>Yes, most of the time<br>Only in select cases<br>No<br>I do not perform SI joint injections
11	What is your preferred injectate for SI joint injections? (Select one)	I do not perform SI joint injections<br>Corticosteroid with local anesthetic<br>Local anesthetic only<br>Platelet-rich plasma (PRP) or other regenerative therapies<br>Other (please specify)
12	Which corticosteroid do you most commonly use? (Select one)	I do not perform SI joint injections<br>Methylprednisolone<br>Triamcinolone<br>Betamethasone<br>Dexamethasone<br>Other (please specify)
13	In patients with both SI and facet pain, which do you treat first? (Select one)	SI joint<br>Facet<br>Whichever is more symptomatic<br>Other (please specify)
14	What volume of injectate do you typically use for therapeutic steroid injections? (Select one)	<1 mL<br>1–2 mL<br>>2–3 mL<br>>3–5 mL<br>>5 mL<br>I do not perform steroid injections
15	What volume of injectate do you use for diagnostic blocks (no steroid)? (Select one)	<1 mL<br>1–2 mL<br>>2–3 mL<br>>3–5 mL<br>>5 mL<br>I do not perform diagnostic SI joint injections
16	Where within the SI joint do you typically inject? (Select one)	Inferior third<br>Middle third<br>Superior third<br>Varies by anatomy or imaging<br>I do not perform SI joint injections
17	How often do you observe a true arthrogram? (Select one)	I do not perform SI joint injections<br>Always<br>Frequently<br>Occasionally<br>Rarely<br>Never

18	Do you use lateral fluoroscopy to confirm placement? (Select one)	I do not perform SI joint injections<br>Always<br>Frequently<br>Occasionally<br>Rarely<br>Never
19	Do you ever see motor block (e.g., patient unable to ambulate) after SI injection? (Select one)	I do not perform SI joint injections<br>Frequently<br>Occasionally<br>Rarely<br>Never
20	Approximately how often do you perform unilateral vs. bilateral injections? (Select one)	I do not perform SI joint injections<br>Mostly unilateral<br>Mostly bilateral<br>About equal
21	How many serious permanent complications have you encountered from SI steroid injections? (Select one)	I do not perform injections<br>0<br>1<br>2<br>3-5<br>5-10<br>>10
22	How often do you perform SI joint RFA? (Select one)	I do not perform SI joint RFA<br>Frequently<br>Occasionally<br>Rarely
23	If not performing RFA, why? (Select one)	Insurance barriers<br>Prefer SI joint fusion<br>Not trained<br>Do not believe it's effective<br>Other (please specify)
24	What settings do you use for RFA? (Select one)	I do not perform RFA<br>Bipolar<br>Monopolar<br>Pulsed<br>Cooled<br>Other (please specify)
25	What technique do you use for RFA? (Select one)	I do not perform RFA<br>Palisade<br>Peri-foraminal<br>Other (please specify)
26	How often does RFA provide >50% relief for >6 months? (Select one)	I do not perform RFA<br>>75%<br>50–75%<br>25–50%<br><25%
27	How many serious permanent complications have you encountered from SI RFA? (Select one)	I do not perform RFA<br>0<br>1<br>2

		3-5<br>5-10<br>>10
28	Do you personally perform SI joint fusion? (Select one)	Yes<br>No
29	If no, would you consider doing it in the future? (Select one)	Not applicable – I personally perform SIJ fusions.<br>Yes<br>No<br>Unsure
30	If you do not personally perform SI joint fusion, how do you typically manage patients who may be candidates for the procedure?	Not applicable – I personally perform SIJ fusions.<br>I refer patients to another physician or surgeon for SIJ fusion.<br>I continue to manage these patients conservatively (e.g., injections, therapy, medications).<br>I discuss surgical options but do not make formal referrals.<br>Other (please specify)
31	How often do you perform or refer for fusion? (Select one)	Frequently (>5/month)<br>Occasionally (1–5/month)<br>Rarely<br>Never
32	Which SI fusion approaches do you have experience performing? (Select all that apply)	I do not have experience with SIJ fusion procedures<br>Lateral<br>Posterior<br>Posterolateral<br>Other (please specify)
33	Which fusion approach do you prefer? (Select one)	Lateral<br>Posterior<br>Posterolateral<br>All are equally effective<br>No preference / Unsure
34	How effective is SI fusion in your opinion? (Select one)	Very effective<br>Moderately effective<br>Minimally effective<br>Ineffective<br>Unsure
35	Which is the better long-term option for SI joint pain in your opinion? (Select one)	RFA<br>SI fusion<br>Depends on the case<br>Unsure
36	How many serious permanent complications have you encountered from SI joint fusion? (Select one)	I do not perform fusion<br>0<br>1<br>2<br>3-5<br>5-10<br>>10
